# Elimination kinetics of diisocyanates after specific inhalative challenges in humans: mass spectrometry analysis, as a basis for biomonitoring strategies

**DOI:** 10.1186/1745-6673-6-9

**Published:** 2011-03-29

**Authors:** Lygia T Budnik, Dennis Nowak, Rolf Merget, Catherine Lemiere, Xaver Baur

**Affiliations:** 1 Institute for Occupational Medicine and Maritime Medicine (ZfAM), University Medical Center, Hamburg-Eppendorf, Hamburg, Germany; 2 Institute and Outpatient Clinic for Occupational, Social and Environmental Medicine, Ludwig-Maximillian-University Munich, Germany; 3 Institute for Prevention and Occupational Medicine of the German Social Accident Insurance, Institute of the Ruhr-University (IPA), Bochum, Germany; 4 University de Montréal, Departement of Medicine, Centre de recherche de l'Hôpital du Sacré-Coeur de Montréal, Montréal, Canada

**Keywords:** isocyanates, biomonitoring, biological monitoring, exposure assessment, occupational asthma, hypersensitivity pneumonitis, specific inhalation challenge

## Abstract

**Background:**

Isocyanates are some of the leading occupational causes of respiratory disorders, predominantly asthma. Adequate exposure monitoring may recognize risk factors and help to prevent the onset or aggravation of these aliments. Though, the biomonitoring appears to be most suitable for exposure assessment, the sampling time is critical, however. In order to settle the optimal time point for the sample collection in a practical biomonitoring approach, we aimed to measure the elimination of isocyanate urine metabolites.

**Methods:**

A simple biomonitoring method enabling detection of all major diamine metabolites, from mono-, poly- and diisocyanates in one analytical step, has been established. Urine samples from 121 patients undergoing inhalative challenge tests with diisocyanates for diagnostic reasons were separated by gas chromatography and analyzed with mass spectrometry (GC-MS) at various time points (0-24 h) after the onset of exposure.

**Results:**

After controlled exposures to different concentrations of diisocyanates (496 ± 102 ppb-min or 1560 ± 420 ppb-min) the elimination kinetics (of respective isocyanate diamine metabolites) revealed differences between aliphatic and aromatic isocyanates (the latter exhibiting a slower elimination) and a dose-response relationship. No significant differences were observed, however, when the elimination time patterns for individual isocyanates were compared, in respect of either low or high exposure or in relation to the presence or absence of prior immunological sensitization.

**Conclusions:**

The detection of isocyanate metabolites in hydrolyzed urine with the help of gas chromatography combined with mass spectrometric detection system appears to be the most suitable, reliable and sensitive method to monitor possible isocyanate uptake by an individual. Additionally, the information on elimination kinetic patterns must be factored into estimates of isocyanate uptake before it is possible for biomonitoring to provide realistic assessments of isocyanate exposure. The pathophysiological elimination of 1,6-hexamethylene diamine, 2,4-diamine toluene, 2,6-diamine toluene, 1,5-naphthalene diamine, 4,4'-diphenylmethane diamine and isophorone diamines (as respective metabolites of: 1,6-hexamethylene diisocyanate, 2,4-toluene diisocyanate and 2,6 toluene diisocyanate, 1,5-naphthalene diisocyanate, 4,4'-diphenylmethane diisocyanate and isophorone diisocyanates) differs between individual isocyanates' diamines.

## Background

The lungs represent the first line of defense against challenges by a variety of environmental dusts, gases, fumes and vapors. Agents like diisocyanates have the capacity to irritate, sensitize and induce toxic effects in the respiratory tract [1,2], depending on the concentration and duration of exposure as well as their physicochemical properties. Exposure to isocyanates occurs either during manufacture (i.e. of foams, elastomers) or during application of spray paints, varnishes, surface coatings, hardeners or binders. Isocyanates, which are characterized by the highly reactive N = C = O groups, are one of the most frequent causes of occupational asthma and can also elicit hypersensitivity pneumonitis and accelerated lung function loss [3-7].

Clinical diagnosis and the differential identification of isocyanates as the cause of work-related disorders are often difficult because of multiple exposures (i.e. to HDI and to MDI/TDI during spray painting) [1,3,7]. Exposure monitoring may recognize risk factors for disease development and help to prevent the onset or aggravation of disease. Efficient methods are needed to improve both primary preventive measures and the surveillance of exposed workers. The increasing use of isocyanates in industrial applications worldwide has increased the likelihood of exposure events in both production plants and during transport. A reliable measurement system of incidental exposure would also benefit bystanders and members of the general public, as well.

Contamination of the air provides the major route for isocyanate uptake, but the pattern of exposure cannot be fully characterized by simply monitoring air contamination. Heavy work increases physical demands and ventilation, exacerbating the degree of incorporation. Absorption through the skin, ingestion and individual differences in metabolism should also be considered [8-11]. Furthermore, the measurement of isocyanate levels in air is complicated [12] by the various physical states of isocyanates, as they may occur as gases or aerosols (in particles or droplets of various sizes). Thus, biomonitoring appears to be most suitable tool for assessing the various exposure scenarios. Feasible analytical methods for the detection of individual isocyanate metabolites in urine have been described [13-17]. Important data for the design of biomonitoring strategies is largely absent, however, with the exact time point of sampling being especially critical. In order to develop guidelines for adequate exposure control in future, biomonitoring based on standardized methods and ascertained kinetic data is needed.

Stable and reproducible amounts of isocyanates can only be obtained under experimental conditions and, in order to understand the excretion patterns of isocyanate metabolites, the use of a controlled isocyanate atmosphere is essential. The aim of this study was to use the data from such controlled studies to estimate the elimination kinetics of the most widely used isocyanates.

## Methods

### Study subjects

The 121 patients involved in this study were referred to the four outpatient clinics by general physicians, the workers compensation boards or statutory accidental insurance institutions for an extensive occupational asthma diagnosis [18]. The study was approved by the respective Institutional Ethics Committees (to XB in Hamburg; to DN in Munich; to RM in Bochum and to CL in Montreal). All participants gave written informed consent. All subjects had previous occupational exposure to isocyanates (0.3-10 years): 110 males and 11 females with a median age of 45 (20 to 60) years. All subjects reported prior or current work-related respiratory symptoms (shortness of breath and wheezing). 50 (42%) were non-smokers, 58 (48%) were ex-smokers and 12 (10%) were current smokers. 44 (37%) were defined as atopic after skin prick testing with common environmental allergens. The prevailing respiratory symptoms and medical and occupational histories were assessed by physician interviews. Serum creatinine concentrations were within the normal range and none had evidence of renal or hepatic disorders. The gold standard for verifying isocyanate-induced asthma is a specific inhalation challenge (SIC), which can only be performed in a few highly specialized centers in the world [7]. All 121 subjects underwent SIC by several isocyanates (HDI, MDI, TDI, NDI or IPD) in one of the four participating centers. A period of at least three days without known occupational exposure was kept in each case prior to the investigation. 30 subjects (25%) of the study group showed positive SIC results (defined as an asthmatic reaction with a fall of FEV_1 _≥ 20%), 42 subjects (35%) showed bronchial hyperresponsiveness (NSBHR) as evaluated by methacholine challenge testing, according to the centers definition. 17 subjects (14%) had specific IgE antibodies to the particular isocyanate. During follow-up, 25% of the patients were removed from their workplace exposure and 75% underwent or are currently undergoing exposure control.

### Isocyanate exposure

All patients underwent SIC using the isocyanate used at their workplaces [19]. They were exposed to the airborne isocyanate with moderate working load (in Hamburg and Munich) in c. 10 m^3 ^exposure chambers where a fan system ventilated the air mixture at a rate of 6.5 m^3^/min (Hamburg, Munich, Bochum) or with a closed-circuit apparatus (Montreal) [1]. The generation of diisocyanate (HDI, MDI, TDI, NDI or IPDI) standard atmospheres was performed with gas-liquid permeation. In detail, permeation tubes were placed in a generation flask containing about 20 mL isocyanates (≥99%) containing either IPDI (in case of IPDI a mixture of cis- and trans-isomers), 4,4'-MDI, 2,4-/2,6-TDI, 1,6-HDI or 1,5-NDI. Briefly, the solution was heated to either 60°C (HDI, IPDI), 80°C (TDI) or to 145°C (MDI) on a heating block generating an isocyanate-enriched atmosphere under a constant pressure of 1.2 L/min. Isocyanate concentrations in the exposure chamber were monitored with a gas monitoring device system instrument (MDA scientific 7100, Honeywell, Zellweger, Hamburg, Germany). Calibration was performed as recommended by the manufacturer. Additionally, a part of the isocyanate air samples was measured with the HPLC filter extraction method. The relative humidity was 35-50% and the temperature 20-25°C as measured by thermo-hydrometer. Air samples were measured in 2 min intervals by the MDA scientific monitor inside the exposure chambers. Subjects were exposed to the respective isocyanate (i.e. HDI, TDI, MDI, NDI or IPDI) at concentrations between 0.5 and 30 ppb for 0 to 120 minutes. The averaged cumulative median concentration was 5.5 ± 5.1 ppb (see additional file 1, 2, 3 and 4 for details on the method). For data analysis, the study subjects were divided into two exposure groups: low 3.1 ± 1.2 ppb/max. 120 min, or high 13 ± 7 ppb/max. 120 min (shown as gemometric mean±SD). The inhalative uptakes were estimated as pulmonary ventilation exposure level × duration of exposure. The calculated isocyanate load was 496 ± 103 ppb-min (for the low exposure group) and 1569 ± 420 ppb-min (for the high exposure group). FEV_1 _was measured before exposure, in 10 min intervals within the first hour, then every hour until 6 h as well as 24 h after exposure. The urine samples from all patients were collected, according to the settled sampling protocol (see also additional files 1, 2, 3 and 4), at various time points starting from the beginning of the challenge (0 point) up to 24 h, for each person at the given time points. To deliver spot urine samples the patients were given sterile 100 mL polyethylene plastic containers and were asked to wash the hands before voiding to avoid dust failing into the sampling container (e.g. from cloth and skin), The samples were placed in a cool box and send to the analyzing laboratory; aliquots were prepared after vigorous shaking of the sample and were immediately frozen (-20°C).

### Analysis of isocyanate metabolites

**Figure 1:**
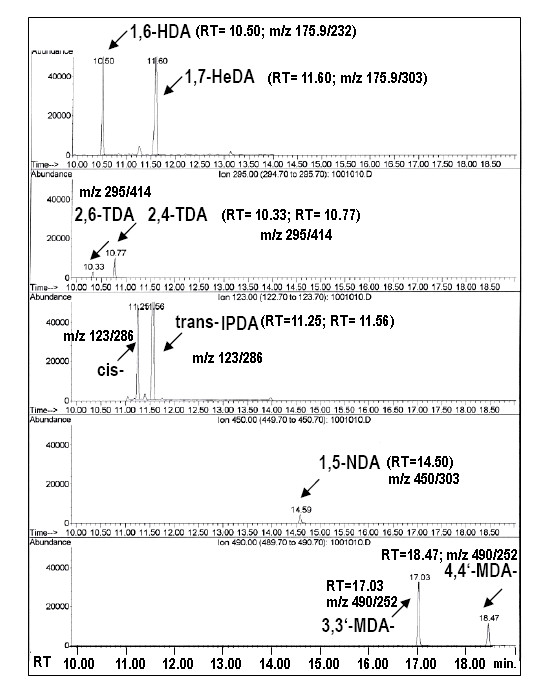
**The GC-MS analysis of isocyanate diamine-metabolites**. Urine samples were subjected to strong acid hydrolysis, separated with gas chromatography (GC) and the individual isocyanate diamines were detected with mass spectrometry (MS), as described in methods. Data show the individual retention time (RT) points (after the GC column separation) and their respective mass/charge (m/z) data (MS detector) with the individual target und qualifier ions allowing to recognize the following metabolites: 1,6 HDA (used to detect 1,6-HDI exposure), 2,4-TDA, 2,6-TDA (to detect 2,4 and 2,6-TDI exposure), cis- and trans- IPDA (to detect exposure to isophorone diisocyanates), 1,5-NDA, (to detect exposure to 1,5 NDI), 4,4'-MDA (to detect exposure to 4,4'-MDI). Additionally, 1,7-HeDA and 3,3'-MDA were used as internal control standards.

The determination of urine metabolites was based on our previously published methods for the single HDA measurements [17] and a single MDA measurement-method published by other group [20] taking advantage of the known biotransformation of isocyanates to respective biological amines and the detection of the parent aromatic amines after acid hydrolysis of urine samples [16,20,21]. The released aromatic amines were separated by gas chromatography and detected by mass spectrometry (GC-MS). The method was modified to perform simultaneous analysis of the metabolites of the following occupational isocyanates in urine: 1,6-HDI, 4,4'-MDI, 2,4-TDI, 2,6-TDI, 1,5-NDI as well as the metabolites of cis- and trans IPDI isomers (Figure 1), thus allowing the monitoring of isocyanate co-exposure mixtures.

It has been recognized earlier that acid hydrolysis yields higher amine values (i.e. 6.5 times higher MDA values were detected). Not only free isocyanate amines, but also additionally bound isocyanates and the conjugates thereof can be detected by this hydrolysis-based method. The acid hydrolysis splits many possible conjugates which might be present in urine like mercapturic acid, glucoronic acid as well as acetyl-/diacetyl isocyanate diamines to corresponding MDA, HDA, TDA, NDA and IPDA. For the current analysis, all patient samples, standards and controls were subjected to strong acid hydrolysis to yield the respective amines: 6 M HCl was added to 2 mL urine, which was hydrolyzed at 100°C for 12 hours, the samples were chilled and made basic with saturated NaOH. The samples were extracted with toluene; after the phase separation two mL dried organic phase by Na_2_SO_4 _were used in derivatization by adding 25 μL pentafluoropropionic anhydride. The vials were closed tightly and shaken for 1 min. The derivatization was stopped by adding 3 mL 1 M phosphate buffer (pH 7.5) and was shaken for 10 min. After centrifugation, the organic phase was supplemented with 100 μL n-decane as keeper and then evaporated with nitrogen to a residual volume of about 100 μL. Two μL of this solution containing the amide derivative were analyzed by GC-MS in a selected ion-monitoring mode on a Agilent mass spectrometry detector MSD HP 5973 connected to a gas chromatograph HP 6890, equipped with an auto-sampler. The separation was performed on a capillary column HP-5MS (30 m × 0.25 mm) with a film thickness of 0.5 μm. The column was held at 100°C for 2 min, ramped at 10°C/min to 280°C. Injections were performed in the split less mode under helium at a flow rate of 2.0 mL/min. Under these conditions, the retention times (RT) for cis-IPDA and trans-IPDA were 11.25 min and 11.56 min, respectively; the specific ions m/z (mass/charge) was 123 (m/z for target ion) and 286 (m/z for qualifier), respectively; for 4,4' MDA the RT was 18.47 min and m/z was 490/252; for 2,4- und 2,6-TDA: 10.33 min and 10.77 min (with m/z 295/414); for 1,6-HDA, 10.50 min (with m/z 175.9/232) and for 1,5-NDA 14.50 min (with m/z 450/303); The RT for 1,7-HeDA was 11.60 min (m/z 175.9/303) and for 3,3'-MDA: 17.03 min (m/z 490/252). The method distinguishes the following isocyanate amines (with the respective instrumental detection limits as shown): 2,4-TDA (0.1 μg/L), 2,6-TDA (0,15 μg/L); 4,4'-MDA (0.1 μg/L); 1,6 HDA (0.5 μg/L); 1,5-NDA (0.5 μg/L)and both isoforms of IPDA, (0.5 μg/L). The 1,7-HeDA and 3,3'-MDA were used as internal standards (to determine the recovery). For interpretation of the data, the peak areas of individual analyzed amines were divided by the peak areas of individual standards. Using this quotient the amine concentrations were estimated with standard curves for each individual isocyanate-amines' run in parallel. Analytical standards for each individual diamine were used to prepare standard calibration curves (7 points). The quantifications were achieved by comparison with these calibration curves (prepared for 1,6 HDA, 2,4-TDA, 2,6-TDA, 4,4'-MDA, 1,5-NDA, both IPDA isomers) in the range of 5 to100 μg/L for each metabolite; additionally; external positive and negative controls were measured within the same analytical step (see Table S2). The analytical limits of detection (LODs) were calculated according to the formula: yB + 3 * sB and were 0.2 μg/L for 2,4-TDA, 0.2 μg/L for 2,6-TDA, 0.3 μg/L for 4,4'-MDA, 1.0 μg/L for 1,5-NDA, 1.0 μg/L 1,6 HDA and 1.0 μg/L for both IPDA isoforms. The levels of the measured isocyanate diamines varied from <0.1 μg/L to 250 μg/L for the time points 0-24 h after the onset of the inhalation challenge. We assessed the method for reproducibility, linearity and sensitivity. Control set points prepared from calibrated standards (see Table S1 for the failure ranges) and control urine samples from non-exposed (5-20 volunteers) and control urine samples from exposed subjects were used as additional internal laboratory controls. The urine samples from non-exposed subjects were below the LOD and the positive controls did not show cross-reactivity (see Table S2 for representative data). All urine values were creatinine-corrected for each sample (the isocyanate concentrations were expressed in μg per g of creatinine). Urinary creatinine was determined in grams per liter (g/L) using HPLC. The method involves the pretreatment of the samples with trichloracetic acid and centrifugation followed by the isocratic separation of compounds on a μ-Bondapak C18 column using a mobile phase consisting of 1.25 mmol/L tetrabutylammonium phosphate (see also additional files 1, 2, 3 and 4 for further details of the methods, validation and controls and materials).

### Data analysis

The excretion of the isocyanate diamines was expressed as median values ± SD (standard deviation) of the respective amine, per g creatinine over the individual time periods. Each sample analysis was performed twice. The data has been divided into low (L) and high (H) exposure groups with 496 ± 103 ppb-min and 1569 ± 420 ppb-min (mean ± SD), respectively. The averaged cumulative mean exposure was calculated for all isocyanates and was used to estimate the general excretion times for each individual isocyanate. To correlate the differences in the excretion times for the respective isocyanate amines between the various groups, the data were sampled using the Pearson approximation method to perform the correlation analysis (the correlation coefficient was calculated to show possible differences between the exposure groups at various time periods after exposure). Geometric means were calculated from the data comprising all groups to estimate the average elimination time patterns for the individual isocyanate diamines. The data analyses were performed with GraphPAD Prism-Software (GraphPad Software Inc., San Diego, CA).

## Results

**Figure 2:**
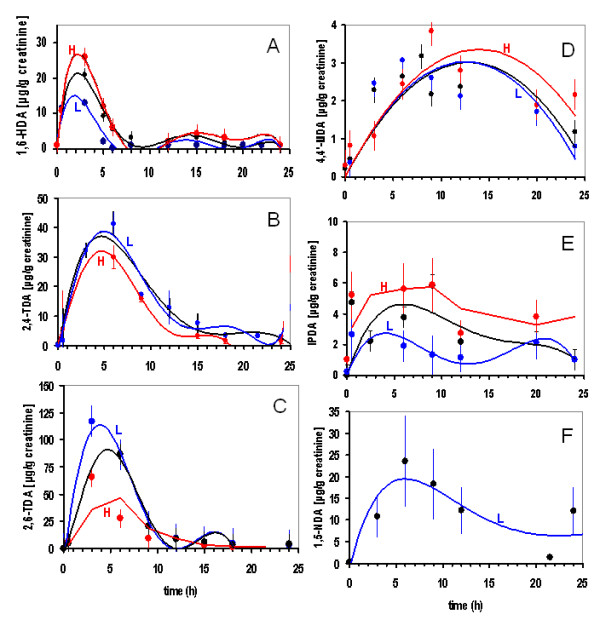
**Elimination kinetics for isocyanate-diamines: 1,6-HDA (A), 2,4-TDA (B), 2,6-TDA (C), 4,4'-MDA (D), sum of the cis- and trans-IPDA isomers (E) and 1,5-NDA (F) in urine of patients after inhalation challenge with either 1,6 -HDI (n = 55), 2,4-TDI (n = 18), 2,6-TDI (n = 18), 4,4'-MDI (n = 36), IPDI (n = 9) or 1,5-NDI (n = 3), respectively**. Spot urine samples were voided by the patients at various time points (the collection times are shown on the × axis) after the controlled exposure (0-24 h). The data points on the Y axis represent median diamine values (expressed as μg/g creatinine) with standard deviations for the patient samples detected with mass spectrometry (analysed against analytical standards for each individual diamine). The trend curves are shown for the low, 496 ± 103 ppb-min (blue, L) as well as high, 1560 ± 420 ppb-min (red, H) exposure groups (see additional files 1, 2, 3 and 4 for details on patient exposure and sampling). The geometric mean was calculated for the cumulative values from all patients to estimate the excretion time points for each individual isocyanate and to calculate the overall trend patterns (black lines).

The median values calculated for each individual time point and the respective isocyanates are shown in the Figures 2A-F. The data was used to estimate the excretion peaks and to calculate the elimination half-lives for low and high exposure groups (). Figure 2A shows the mean values for 55 workers exposed to 1,6-HDI; the 1,6-HDA excretion levels demonstrate a major peak at 2 h and a second smaller one 15 h after the onset of the inhalative challenges, giving a calculated excretion half time of 2.5 h. Figure 2B/C shows the elimination times for the metabolites of the two aromatic isocyanates (2,4-/2,6-TDI). The 2,4-TDA peaked at 4.1 h and 2,6-TDA at 4.8 h, the estimated half time for TDA was 6 h (n = 18). It is known that the respective industrial products represent a mixture of 2,4- and 2,6-TDA which is used at the majority of workplaces. The excretion time for 4,4'-MDA (n = 36) is given in Figure 2D, and shows a peak at 14 h after the exposure. Figure 2E indicates that the urinary excretion of IPDA peaked at 5.6 h after exposure. The complete elimination of IPDA in the urine was still not reached after 24 h (see below). It has to be noted that the elimination patterns for MDA and IPDA did not show as clear excrection peaks as for TDA or HDA.The elimination kinetics for 1,5-NDA is shown in Figure 2F. Given the reservations arising from the small size of the group with NDI exposure (n = 3), the excretion of NDA peaked at 6.0 h with an additional late peak at >48 h.

Elevated peaks could be seen for the higher exposure groups for 1,6-HDA (with r = 0.86, when the elimination kinetics trends were compared between the low and high exposure groups), 4,4'-MDA and IPDA, but not for 2,4-TDA and 2,6-TDA. For all isocyanate diamines there was a small non-significant shift of excretion for all high exposure groups (as compared to the low exposure groups). This might indicate slightly slower elimination kinetics. A slight shift to longer time periods is evident when comparing the excretion of 4,4'-MDA and IPDA metabolites between the low and high exposure groups (r = 0.7, r = 0.7 elimation trend-patterns for 4,4'-MDA and IPDA, respectively).

Across the individual patient groups, the isocyanate metabolites show similar excretion kinetics patterns. Neither the SIC outcome, the NSBHR nor immunological parameters appear to influence the pathophysiological elimation of individual metabolites. For patients showing either a positive or negative SIC reaction (when the elimimation kinetics were compared between patients with positive or negative SIC reaction) or for patients with or without specific IgE antibodies and confirmed asthma diagnosis, there were no discernable changes in the excretion pattern (no statistically significant differences in r values between the individual groups). See also Figures S1a, S1b in additional file 4 for examples with individual patients.

## Discussion

The sensitive and specific assessment of exposure to airborne agents is a precondition for effective prevention measures and health risk assessment. Air monitoring can be a problem because isocyanate aerosols and simultanous exposures to more than one isocyanate, frequently present in the workplace, are not adequately measured by many routine devices [22]. It has been shown that isocyanate exposure can occur despite respiratory protective equipment, and skin absorption or ingestion also having to be considered [8]. Previous studies have shown that the detection of isocyanate-derived (di-) amines in hydrolyzed urine is the most suitable, acceptable and sensitive method for monitoring potential individual isocyanate exposures [23-26]. Earlier studies provided some evidence that the urine excretion time may differ for individual isocyanates [24,26,27]. We corroborated the differences in excretion kinetics for different isocyanates and have established the elimination patterns for all major diisocyanates at different exposure concentrations. When looking closer at different isocyanates, it became obvious that the aliphatic isocyanate 1,6-HDI has a shorter excretion time than aromatic isocyanates (4,4'-MDI, 2,4-/2,6-TDI). Notably, aromatic MDA, NDA and cycloaliphatic IPDA were not completely eliminated after 24 h. After pulmonary absorption of 2,4- and 2,6-TDI, the majority had been excreted in urine 6 h after the end of exposure [23,28].

According to other studies, additional slowly generated TDA fragments were released into urine over days [28,29]. Other groups could not monitor any longterm release of TDA into the urine [25]. We observed the major excretion peak at 4.1 h and 4.8 h (for 2,6- and 2,4-TDA); the majority of the TDA appeared to be eliminated after 24 h. At high exposure levels, the TDA was eliminated more slowly with a half time of 6 h.

It has to be noted however that the patients were exposed to a mixture of 2,4-/2,6-TDI, which might influence the elimination of a single diamine, a greater exposure group is necessary to prove this hypothesis. Unfortunately, in many studies only pre- and post-working shift data are provided. This may lead to misinterpretation of the actual exposure since only 15-20% of the residual 2,4- and 2,6-TDA is found after 8 h.

In many industrial workplaces, exposure to several isocyanates may take place simultaneously and no information is available about how the different isocyanates are metabolized when the atmosphere contains a mixture of several isocyanates, such as e.g. during thermal degradation of polyurethanes. Other authors have identified MDA in pooled urine samples after exposure to MDI from thermal breakdown [15,20,30]. A high variability in TDA and MDA concentrations was described in urine during and between workdays [31-33], but information on the elimination half-times of MDA or NDA was not available as yet. We observed clear excretion peaks between 12-14 h after the end of exposure, revealing urinary elimination of MDA that is significantly slower than for other isocyanate amines. It was also evident that the excretion was not complete after 24 h. We observed similarly slow elimination rates for another aromatic diisocyanate, 1,5-NDI, in another investigation of workplace exposure, with elimination times over 2-5 days in 6 workers (data not shown). We have estimated the excretion half-life for IPDA to be 4-5.5 h (for low and high exposure groups, respectively). In an earlier IPDI exposure study, the urinary elimination half-times of IPDA excretion seemed to be slightly faster, reaching the half-time of excretion values between 1.7-4.3 h for subjects not previously exposed [34].

Our findings indicate that there is a clear difference in the excretion kinetics for individual isocyanates. Thus the measurements obtained after a working shift may falsely estimate the degree of exposure, especially for the aliphatic HDA with extremely short excretion times or aromatic isocyanates (i.e. MDA, NDA) with their longer excretion times. Interestingly, increasing the isocyanate load during the exposure challenge did not change the overall kinetic patterns, rather inducing a more prolonged horizontal shift (i.e. MDA, IPDA). There were only small differences in the excretion kinetics for the low and high exposure groups of investigated subjects when the individual peak hights were compared. It cannot be excluded that the isocyanate may metabolize differently if air concentrations are higher than those in this study.

Neither prior isocyanate exposure, bronchial hyperresponsiveness nor immunological sensitization to isocyantes were associated with changes in the pattern of the elimination kinetics. It had been proposed that chronically exposed workers might metabolize isocyanates differently than volunteers without prior exposure [33]. We cannot exclude this, but we found similar elimination kinetics for individual diisocyanates despite the different occupational pre-exposure histories of the subjects, their clinical status and different demographic and geographic origins.

It is likely that the same metabolizing enzyme or various (produced) adducts influence the elimination kinetics. The molecular pathomechanism of the isocyanate transport to an affected organ, the development of the disease and its elimination from the body are largely unknown for humans. It is assumed that the isocyanates are hydrolysed to their respective amines and further oxidized by the cyclooxygenase, CYP, to N-hydroxyarylamine and to nitroso compounds with glutathione as an important vehicle [14,15,35], with enzyme polymorphisms presumably having an effect. The short lifetime of isocyanate amines means that urinary sampling is often too late, limiting their applicability as a useful biomarker of recent exposure. To monitor longterm exposure, other biomarkers could be considered, with the measurement of DNA- and/or protein adducts offering promise. Novel industrial isocyanates may need modifications of the currently proposed methods for monitoring exposures, especially if they differ substantially from the usual chemical entities.

A major advantage of biomonitoring urinary metabolites is the provision of a measurement that reflects the total dose of isocyanates absorbed by the body by all routes. The simultaneous screening of the urine metabolites of aromatic, aliphatic and cycloaliphatic isocyanates enhances the probability of detecting previously unappreciated exposure. Using this method, we performed the biomonitoring of a group of 55 car industry workers and detected a high exposure to a totally unexpected isocyanate source, which proved to be a novel paint formulation recently introduced into the working process [36].

## Conclusions

The detection of isocyanate metabolites in hydrolyzed urine with the help of GC-MS appears to be the most suitable, reliable and sensitive method to monitor possible isocyanate uptake by an individual. The simplified sample collection of spot urine, increases both the acceptance and penetrance of monitoring for both patients and physicians. Simultaneous screening within the same analytical step enables the effective monitoring of mixtures of monoisocyanates, diisocyanates and oligoisocyanates, which are the prevailing substances in various industrial settings. Since excretion kinetics patterns vary for different isocyanates, these kinetics must be considered in planning biological monitoring in which urinary elimination is used as an estimate of uptake. Two different sampling time points might be appriopriate for most work settings.

## Abbreviations

1,6-HDI: (1,6-hexamethylene diisocyanate); 2,4-TDI, 2,6-TDI: (2,4- and 2,6 toluene diisocyante); 1,5-NDI: (1,5-naphthalene diisocyanate); 4,4'- MDI: (4,4'- diphenylmethane diisocyanate; IPDI: (isophorone diisocyanate); IPDA: (Isophorone diamine); 1,6 HDA: (1,6-hexamethylene diamine); 1,7 HeDA: (1,7-diaminoheptane); 2,4-TDA: (2,4-diamintoluene); 2,6-TDA: (2,6-diamintoluene); 1,5-NDA: (1,5-naphthalene diamine); 4,4'-MDA: (4,4'-diphenylmethane diamine); 3,3'-MDA: (3,3'-methylene dianiline); SIC: (specific inhalation challenge); NSBHR: (nonspecific bronchial hyper responsiveness); SPT: (prick test).

## Competing interests

None of the authors has a financial relationship with a commercial entity that has an interest in the subject of this manuscript.

## Authors' contributions

XB planed the study, XB, RM, DN, CL have supervised the specific inhalative challenges, the examination of the patients and diagnosis; LTB was responsible for all laboratory tests; LTB/XB drafted the manuscript, LTB wrote the mansuscript. All the authors have read and approved the final version of the manuscript.

## Supplementary Material

Additional file 1 **Materials**. Click here for file

Additional file 2 **Supplementary data to the methods**. Click here for file

Additional file 3 **Validation data to the GC-MS-method**. Click here for file

Additional file 4 **Examples of individual patient data**. Click here for file
