# Supplementary material for: Elimination kinetics of diisocyanates after specific inhalative challenges in humans: mass spectrometry analysis, as a basis for biomonitoring strategies
Source: J Occup Med Toxicol. 2011 Mar 29;6:9. doi: 10.1186/1745-6673-6-9 (PMC3080353; doi:10.1186/1745-6673-6-9)
Supplement: Additional file 1 — Materials. [file 1745-6673-6-9-S1.DOC]

**Additional file 1. Materials**

Chemicals used were: 96% sulfuric acid, sodium sulphate (Merck, Darmstadt, Germany), toluene, n-decan, IPDA (isophorone diamine), 1,6 HDA und 1,7 HeDA (1,6--hexamethylene diamine, 1,7-diaminoheptane); 2,4-TDA (2,4-diaminotoluene), 2,6-TDA (2,6-diaminotoluene), 1,5-NDA (1,5-naphthalene diamine) 4,4´-MDA (4,4´-diphenylmethane diamine), 3,3´-MDA (3,3´-methylene dianiline), pentafluorpropione acid anhydrid (PFPA) (Sigma-Aldrich, Taufkirchen, Germany), HDI (hexamethylene diisocyanate), MDI (diphenylmethane diisocyanate), TDI (toluene diisocyanate), NDI (naphthalene diisocyanate), IPDI (isophorone diisocyanate (either from Th Geyer, Hamburg, Germany or Merck, Darmstadt, Germany). Isocyanates were of extra pure grade, all other chemicals were of analytical grade.
