# Supplementary material for: Elimination kinetics of diisocyanates after specific inhalative challenges in humans: mass spectrometry analysis, as a basis for biomonitoring strategies
Source: J Occup Med Toxicol. 2011 Mar 29;6:9. doi: 10.1186/1745-6673-6-9 (PMC3080353; doi:10.1186/1745-6673-6-9)
Supplement: Additional file 2 — Supplementary data to the methods. [file 1745-6673-6-9-S2.DOC]

**Additional File 2: Supplementary data to the methods**

**Controlled isocyanate exposure:** To measure the systemic levels of isocyanate-derived amines, urine samples from the 121 patients were taken at various time points (0-24 h) after to HDI, MDI, TDI, NDI or IPDI exposure under laboratory conditions in four different centers (using isocyanates known from the patients work places. The patients collected spoted urine samples as described in the methods (see also the information below). All samples were sent per express to Hamburg, were immediately frozen below -20°C and measured with a validated analytical method described herewith.

**Urine sampling for isocyanate biomonitoring:**

The urine samples from all patients were collected, according to the settled sampling protocol, based on the IUPAC, CDC and WHO rules [1,2,3] at various time points starting from the begin of the challenge (0 to 24h). The WHO settled criteria for the spoted urine sampling and recommended calculation of the urine based biological monitoring data were applied; Urinary creatinine concentrations and specific gravity were measured for each sample for adjusting dilutions.

Variations in urinary analyte concentrations from changing water content in urine were eliminated using urinary excretion rate calculations; thereby the most widely used creatinine adjustment was applied (that involves dividing the analyte concentration, micrograms analyte per gram creatinine). [3]. The common method correlates the metabolite concentrations better with blood concentrations of the parent chemical than other known methods [3].

The major advantage of using urine for biomonitoring analysis is its ease of collection for spot (timed) or grab (untimed, “convenience” samples) urine samples, but not for 24-h urine voids [2,3]. Because 24-h collection can be cumbersome, often resulting in inproper or incomplete collection, the 24-h samples are most commonly wanted by physicians from bed-ridden patients only. Most 8-h shift workers do not like providing 24-h sampling, so that control of sample collection and storage is difficult and hard to verify when the workers are not at the workplace.

The WHO guidelines for valid urine samples for occupational monitoring [5] recommends that if the sample is too diluted (creatinine value <0.3 g/) or too concentrated (creatinine value >3 g/L) urine specimen should be excluded as not acceptable for biomonitoring. Therefore only urine samples within the before-mentioned acceptable range were used in this study.

**Analysis of isocyanate metabolites** (1,6-hexamethylene diamine, 2,4-diamine toluene, 2,6-diamine toluene, 1,5-naphthalene diamine, 4,4´diphenylmethane diamine and both cis and trans isophorone diamine isomers) was performed using GC-MS analysis. All data were adjusted with creatinine for the different urine volumes. Urinary creatinine in each sample was determined in grams per liter (g/L) using HPLC method [4]. The isocyanate concentrations were then expressed in µg per g of creatinine.

The German MAK-Commission (Commission for the investigation of Health Hazards of Chemical Compounds in the Work Area) has recommended [5] the limit value (Biological Workplace Tolerance Value; BAT) for one isocyanate metabolite only: the 4,4’-MDA has BAT-Value of 10 µg/g creatinine (for spotted urine, post shift sample).
