# Supplementary material for: Elimination kinetics of diisocyanates after specific inhalative challenges in humans: mass spectrometry analysis, as a basis for biomonitoring strategies
Source: J Occup Med Toxicol. 2011 Mar 29;6:9. doi: 10.1186/1745-6673-6-9 (PMC3080353; doi:10.1186/1745-6673-6-9)
Supplement: Additional file 3 — Validation data to the GC-MS-method. [file 1745-6673-6-9-S3.DOC]

**Additional file 3: Validation data to the GC-MS-method:**


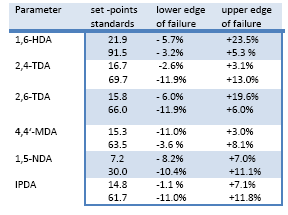


***Table S 1:***

*Control set points (prepared with analytical standards) with failure range*

*All diamine values are given in µg/g creatinine*

***
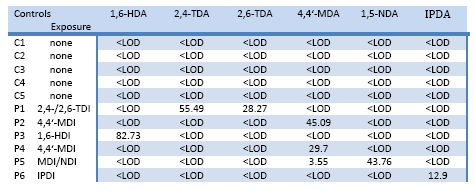
***

***Table S2****: Internal laboratory control samples (negative and positive urine samples from known unexposed and exposed subjects).*

*All diamine values are given in µg/g creatinine.*
