# Supplementary material for: Elimination kinetics of diisocyanates after specific inhalative challenges in humans: mass spectrometry analysis, as a basis for biomonitoring strategies
Source: J Occup Med Toxicol. 2011 Mar 29;6:9. doi: 10.1186/1745-6673-6-9 (PMC3080353; doi:10.1186/1745-6673-6-9)
Supplement: Additional file 4 — Examples of individual patient data. [file 1745-6673-6-9-S4.DOC]

**Additional file 4: Examples of individual patient data:**

Individual patient data were analyzed with respect to the demographic data, lung function, allergy status and the presence of absence of specific antibodies. The supplementary Figs.1a, 1b show exemplified individual data for 4 patients exposed to low isocyanate load. Chosen were 2 examples for 2 individual isocyanates with different elimination kinetics.

***Fig. S1a***

***Elimination kinetic for 4,4´-MDA.*** *Shown are individual data for 2 patients exposed to 4,4´-MDI (both low exposure group). Urine samples were collected at various time points after the SIC exposure (0-24h) and were analyzed as described above.*

Pat. # 1, m, 31ys, smoker (4.5 py), 5.5 ys occupational exposure to MDI, atopy status,

positive NSBHR, positive. MDI-SIC reaction, positive MDI-SPT (skin prick test), presence of

MDI-specific IgE and IgG-antibodies; diagnosis: isocyanate asthma (green line, empty

symbols)

Pat. # 2. m, 33ys, non-smoker, 1.5 ys occupational exposure to MDI, non-atopy status, no NSBHR,

restrictive ventilation pattern, negative MDI-SIC, negative MDI-SPT, no MDI-specific

IgE- or IgG-antibodies; diagnosis: mild restrictive lung disese (blue line, filled symbols)

***Fig. S 1 b***

***Elimination kinetic for 2,6-TDA.*** *Shown are individual data for 2 patients exposed to TDI (both low exposure group).*

*Urine samples were collected at various time points after the SIC exposure (0-24h) and were analyzed as described above.*

Pat.# 3, m, 30ys, no smoker, 9.7 ys occupational exposure to TDI, no atopy status, no NSBHR,

negative TDI-SIC, negative TDI-SPT, no TDI-specific IgE-antibodies, specific IgG-antibodies

present; diagnosis: hypersensitivity pneumonitis (violet line, filled triangles)

Pat.# 4. m, 65 ys, no smoker, 1.5 ys occupational exposure to TDI, atopy status, NSBHR,

positive TDI-SIC, positive TDI-SPT, no TDI-specific IgE- and IgG antibodies, diagnosis: COPD

(orange line, filled squares)

**References to additional files:**

1. R.Cornelis, B. Heinzow, R.F.M: Herber, J. Molin Christiansem, O.M. Paulse, E. Sabbioni, DM Templeton, Y Thpmassen, M. Vahter, O. Vesterberg. International Union of Pure and Appled Chemistry. Clinical Chemistry Division, Commissoon on Toxicology, Working party. Sample collection guidelines for trace elements in blood and urine, 1996, Pure & Appl. Chem. 67: 1575-1608.
2. WHO, Biological Monitoring of Chemical Exposure in the Workplace, 2004, Environmental Health Criteria No. 78, Geneva World health Organisation
3. D. S. Barr, L.C. Wilder; S. P. Caudill, A. J. Gonzaöez, L.L. Needham, J.L. Pirkle. Urine Creatinine Concentrations in the US Population: Implications for Urinary Biological Monitoring Measures. 2005 Environ Health Perspect 113: 192-200
4. H.Müller, HPLC-method fort he simultanous determination of urea, creatinine, and uric acid in serum and urine. Fresenius Z. Anal. Chem. 1988, 332, 464-467 (in German).
5. DFG Commission for the Investigation of Health Hazards of Chemical Compounds in the Work Area, MAK- and BAT--Values. 2010, Vol. 46., Wiley-VCh
